# ProTransition – evaluation of an online-course for professionals to optimize the health care service for young people with mental illness in transition from adolescence to adulthood

**DOI:** 10.1192/j.eurpsy.2022.1791

**Published:** 2022-09-01

**Authors:** E. Koenig, S. Reetz, U. Hoffmann, J.M. Fegert

**Affiliations:** Universityhospital Ulm, Child And Adolescent Psychiatry/ Psychotherapy, Ulm, Germany

**Keywords:** transition psychiatry, e-learning

## Abstract

**Introduction:**

Adolescent transitions to adulthood are a vulnerable phase for the development of mental illnesses. Additionally, there are often disruptions in psychiatric care delivery during the transition phase, potentially leading to a considerable treatment delay with a high risk of early chronification. Thus, the health care system and professionals in both child and adolescent psychiatry and adult psychiatry should give greater consideration to the transition phase.

**Objectives:**

An online-course addressing health care professionals was developed to give in-depth knowledge of “transition psychiatry”, practical guidance and to sensitize for the special challenges and needs of young adults with mental illness. Evaluation focuses on the gain of competences, benefit for practical work and user satisfaction.

**Methods:**

Participants´ estimations and opinions on quality of the online-course, on impact of course participation to their practical work and on their competences regarding transition psychiatry are assessed with an online-survey before starting (t1) and after finishing (t2) the online-course. T1-assessment is already completed with 1924 datasets, t2-assessmend will take place 02/2022.

**Results:**

Analyses of t1-assessment show a high heterogeneity of participants regarding their work background and setting. Special knowledge about mental illnesses during transition and about transition psychiatry, as well as feeling confident in accompanying transition processes is on a medium level. Results of t2-assessment and comparing analyses are expected in March 2022 and will be presented.

**Conclusions:**

There was high interest of the target group in participating in the online-course. Evaluation will show if the online-course is a helpful measure in delievering the necessary education of professionals in transition psychiatry.

**Disclosure:**

No significant relationships.

